# Myocardial recovery following durable left ventricular assist device support

**DOI:** 10.1016/j.xjon.2021.10.005

**Published:** 2021-10-22

**Authors:** Vivek Rao, Filio Billia

**Affiliations:** a Division of Cardiovascular Surgery, Advanced Heart Failure Program, Peter Munk Cardiac Centre, Toronto General Hospital, University Health Network, Toronto, Ontario, Canada; b Division of Cardiology, Advanced Heart Failure Program, Peter Munk Cardiac Centre, Toronto General Hospital, University Health Network, Toronto, Ontario, Canada

**Keywords:** mechanical circulatory support, myocardial recovery, congestive heart failure

**Figure:**
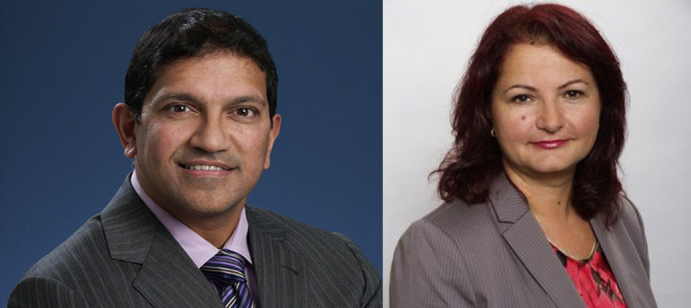
Vivek Rao, MD, PhD, FRCSC, FAHA, and Filio Billia, MD, PhD

**Central Message**

Myocardial recovery in patients supported by durable MCS devices is well recognized. However, predicting which patients will recover and improving the rates of recovery remains challenging.

See Commentaries on pages 6 and 8.

Durable left ventricular assist device (LVAD) support as a bridge to recovery leading to LVAD explantation remains the holy grail of advanced therapies for heart failure. Although pharmacologic therapy using the Harefield Protocol with Clenbuterol (a β_2_ agonist) resulted in device explant in more than two-thirds of patients, these results have not been widely reproducible.^1^^,^^2^

## What is Recovery?

Myocardial recovery can be enhanced, it can be observed to occur naturally, and it can be forced. For the purposes of this Commentary, we will focus on myocardial recovery that occurs in patients with a long-standing history of congestive heart failure who are supported by a mechanical device for at least 90 days.

Although many physicians in the field have observed myocardial recovery following an acute insult, patients often succumbed to multiorgan failure due to the inability of the heart to maintain adequate end-organ perfusion. Several technologies exist to temporarily support circulation until native myocardial recovery occurs. These short-term devices are routinely used to support patients experiencing acute cardiogenic shock secondary to acute myocardial infarction, fulminant viral myocarditis, postpartum cardiomyopathy, and perhaps most commonly postcardiotomy shock. For most of these situations, myocardial recovery is expected and thus a short-term circulatory support device is chosen. Recovery rates for these etiologies is >75% and survival to hospital discharge often exceeds 50%.

**Figure 1:**
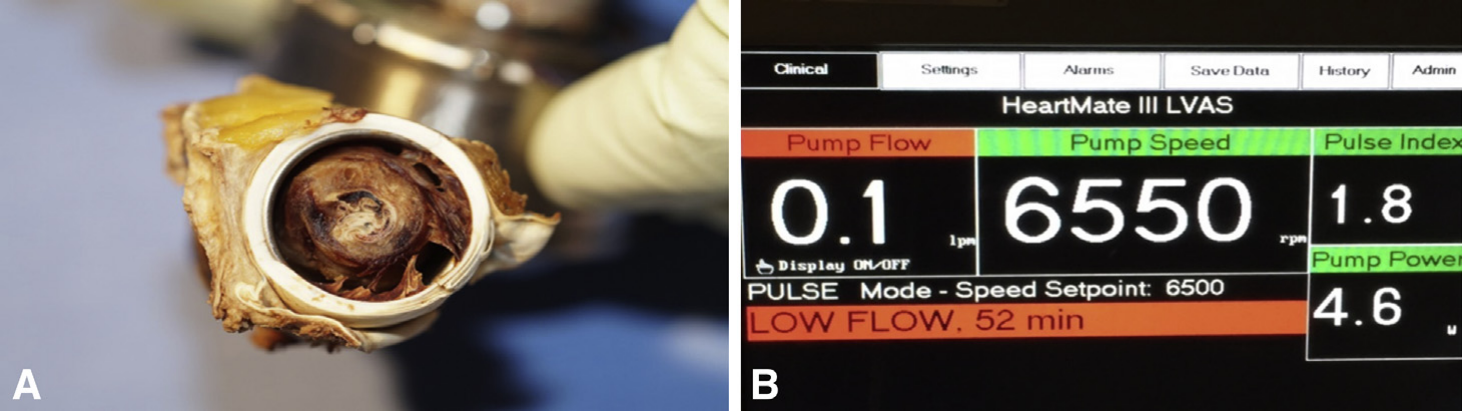
A, An example of outflow graft thrombus between the bend relief and the polyethylene terephthalate graft of a HeartMate 3 (Abbott Cardiovascular, Abbott Park, Ill) left ventricular assist device (*LVAD*). B, The outflow graft compression results in nonsignificant flow from the LVAD as seen by the display monitor.

In contrast, myocardial recovery can sometimes be forced. The most common indication for forced recovery is a refractory infection of the LVAD housing and/or driveline. Complete explant of the device is often the only option in nontransplant-eligible patients and the ability of the native heart to maintain adequate hemodynamic parameters—even with a poor LV ejection fraction—is commonly seen. Similarly, acute or subacute pump malfunction has led to the inadvertent recovery of the native heart because the pump ceases to provide any circulatory support. Figure 1, *A*, illustrates circumferential and compressive clot between the bend relief and the outflow relief graft of a HeartMate 3 (Abbott Cardiovascular, Abbott Park, Ill) LVAD. Our group initially reported that the patient presented with low-flow alarms and eventually displayed no meaningful pump output (Figure 1, *B*).^3^ Although technically this patient's native myocardium recovered to the point where it was able to provide stable hemodynamic parameters in the setting of pump failure, recovery was not deemed to be durable and the patient was subsequently transplanted successfully. Such cases may be thought of as myocardial recovery, but the extent of functional recovery and the durability of this recovery remain questionable.

## Enhanced Recovery

For the purposes of this review, we have intentionally excluded the prior examples of recovery. Rather, we focus on the potential ability of durable LVAD support to enhance reverse remodeling to the extent that functional recovery permits device explant. The concept of enhancing myocardial recovery to facilitate LVAD explant is largely credited to the Harefield group, which published their initial experience with 15 patients in 2006.^1^

All patients received guideline-directed medical therapy until regression of LV size was observed. They were then started on the β-agonist clenbuterol in an attempt to stimulate myocardial metabolism and cellular hypertrophy. Of these 15 patients, 11 displayed sufficient recovery to permit LVAD explant. There was 1 early death within 24 hours of explant and 1 late death at 27 months due to carcinoma. At 60 months of follow-up, the mean LVEF was sustained at 64%.

There are a few important observations from the Harefield experience. Firstly, they excluded patients with ischemic cardiomyopathy who were unlikely to recover. Only 15 of the 24 patients with nonischemic cardiomyopathy completed the pharmacologic protocol yielding an explant rate of 11 out of 24 (46%). The explant rate in those that completed the full protocol was 11 out of 15 and if we exclude the early death, the successful explant rate was 67%. This level of recovery has yet to be duplicated.

In the most recent Interagency Registry for Mechanically Assisted Circulatory Support report, the explant rate in more than 14,600 patients was only 5%. Again, many of these explants were forced due to infection or pump malfunction.^2^ The LVAD Working Group published the results of a multicenter study of myocardial recovery in 2007.^4^ Out of 67 patients recruited to complete the pharmacologic therapy, only 6 displayed sufficient recovery to permit explant. Of note, only 55% of patients in this series had nonischemic cardiomyopathy. Of the 6 explants, 4 presented with acute heart failure and only 2 experienced chronic heart failure. Not surprisingly, those patients who received support during an acute presentation demonstrated sustained recovery of LV function following LVAD explant. In contrast, both patients with chronic heart failure displayed a decline in LV ejection fraction, although there were no clinical sequelae (ie, transplant or new VAD implant).

The Cardiothoracic Surgical Trials Network supported a prospective, randomized trial evaluating the efficacy of concomitant mesenchymal stem cell injection at the time of LVAD implant.^5^ The proposed benefits of stem cell injection remain controversial, but include direct replacement of scar with viable myocardium as well as a potent angiogenic response to cell injection independent of the viability of the injected cells. One hundred fifty-nine patients were recruited into this trial and no patient exhibited sufficient recovery to permit LVAD explant. There were no differences in LV ejection fraction at 6 months and no differences in the ability to tolerate temporary weaning from the LVAD. An unexplained observation from this trial was that patients who received cell injections experienced less mucosal bleeding (17% vs 33%; *P* = .02).

The recently reported Remission from Stage D Heart Failure trial results suggest that >50% of patients with nonischemic cardiomyopathy can undergo successful LVAD explantation after optimization with a standardized pharmacologic regimen using a cocktail of widely available heart failure drugs.^6^ At 2 years following explant, the freedom from transplant or new LVAD was 77%, suggesting that the recovery in myocardial function is durable.

It appears that in carefully selected patients with chronic, nonischemic cardiomyopathy, myocardial recovery can be enhanced with targeted pharmacologic therapy. In those patients who display sufficient recovery to permit VAD explant, the recovery in myocardial function appears durable. However, when placed in context of all patients who receive durable mechanical circulatory support for either acute or chronic heart failure (many of whom have ischemic heart disease), the prevalence of true myocardial recovery remains small.

## Assessing for Recovery

Despite the low overall prevalence of myocardial recovery following LVAD support, we continue to assess all patients in a standardized fashion. Many protocols have been published to assess for myocardial recovery and to predict successful explant.^5^, ^6^, ^7^, ^8^ We start all patients on guideline directed medical therapy. Patients are assessed with transthoracic echocardiograms every 3 months to optimize pump speed, document LV size and function, and to rule out new regurgitant lesions.

Clearly, the need to address significant aortic or mitral insufficiency at the time of LVAD explant increases the perioperative risk of surgery and likely impairs the durability of recovery. Some authors have advocated for routine, repeated invasive hemodynamic studies to optimize LVAD function, and promote recovery.^7^ Although we agree that invasive studies can aid in the management of some patients, we find it impractical to implement these studies in all LVAD recipients at regular intervals.

In our group, we assess LV size and function and perform invasive evaluations only in the case that LVAD explant is contemplated. In that event, the patient is heparinized and LVAD support is minimized, whereas invasive monitoring of arterial blood pressure, central venous pressure, and pulmonary pressures are performed.^8^ If a patient is clinically stable and the hemodynamic parameters support LVAD explant, we proceed to surgery. However, in our experience, only 5% of durable LVAD recipients have exhibited sufficient recovery (with the cutoff of LV ejection fraction >50%) to permit LVAD explant. This is similar to the rates published by the Interagency Registry for Mechanically Assisted Circulatory Support Registry.^2^

## Molecular Markers of Recovery

Heart failure is characterized by cardiomyocyte hypertrophy, expression of the fetal gene program, uncoupling of the G protein coupled receptors driving sympathetic hyperactivity, dysregulation of calcium handling,^9^ disruption of many signaling pathways, and mitochondrial dysfunction.^10^^,^^11^ Guideline-directed medical therapy has been shown to undo the reverse remodeling and cardiomyocyte hypertrophy that is present in patients with heart failure. The ability of mechanical unloading with an LVAD to reduce the degree of cardiomyocyte hypertrophy is controversial. Consistent with LVAD-induced reduction in cardiomyocyte hypertrophy, mechanical unloading also leads to a reduction in the myocardial expression of the fetal gene program. In addition, there is restoration of dysregulated calcium handling and activation of MAPK-ERK signalling cascade with LVAD support.^10^ This is in conjunction with restoration of myocardial energetics with enhanced transcription of genes involved in fatty acid, pyruvate, and glucose metabolism.^12^, ^13^, ^14^, ^15^, ^16^

Transcriptional profiling has been used to provide insights into mechanisms of LVAD-induced reverse remodeling and to identify novel biomarkers of myocardial recovery. In a comprehensive study, only 238 out of 3088 (7.7%) transcripts that were dysregulated in heart failure, were altered with LVAD support.^17^ Of note, genes that were significantly dysregulated with LVAD support were more likely to exhibit further deviation from the nonfailing transcription levels instead of returning toward normal. Of the heart failure dysregulated genes that normalized with LVAD support, several messenger RNAs were enriched in inflammatory and oxidative stress pathways.

In another study, sequence-based transcriptional profiling of LVAD-induced changes in messenger RNA, micro RNA, and long noncoding RNA were examined.^15^ Of all the transcripts examined, long noncoding RNAs exhibited the high level of improvement with LVAD support. Nevertheless, >90% of the transcripts, including coding and noncoding segments, remain persistently dysregulated following LVAD support. It is important to point out that most studies did not account for the heterogeneity of the cell populations used for RNA extraction.

## Surgical Explant

Surgical explant of a durable LVAD can be challenging, particularly in the case that the patient has been supported for >90 days. Forced explant due to pump infection usually necessitates complete excision of all foreign material mandating redo sternotomy. However, explant due to recovery can be accomplished with less-invasive techniques, tailored to the specific device.

**Figure 2:**
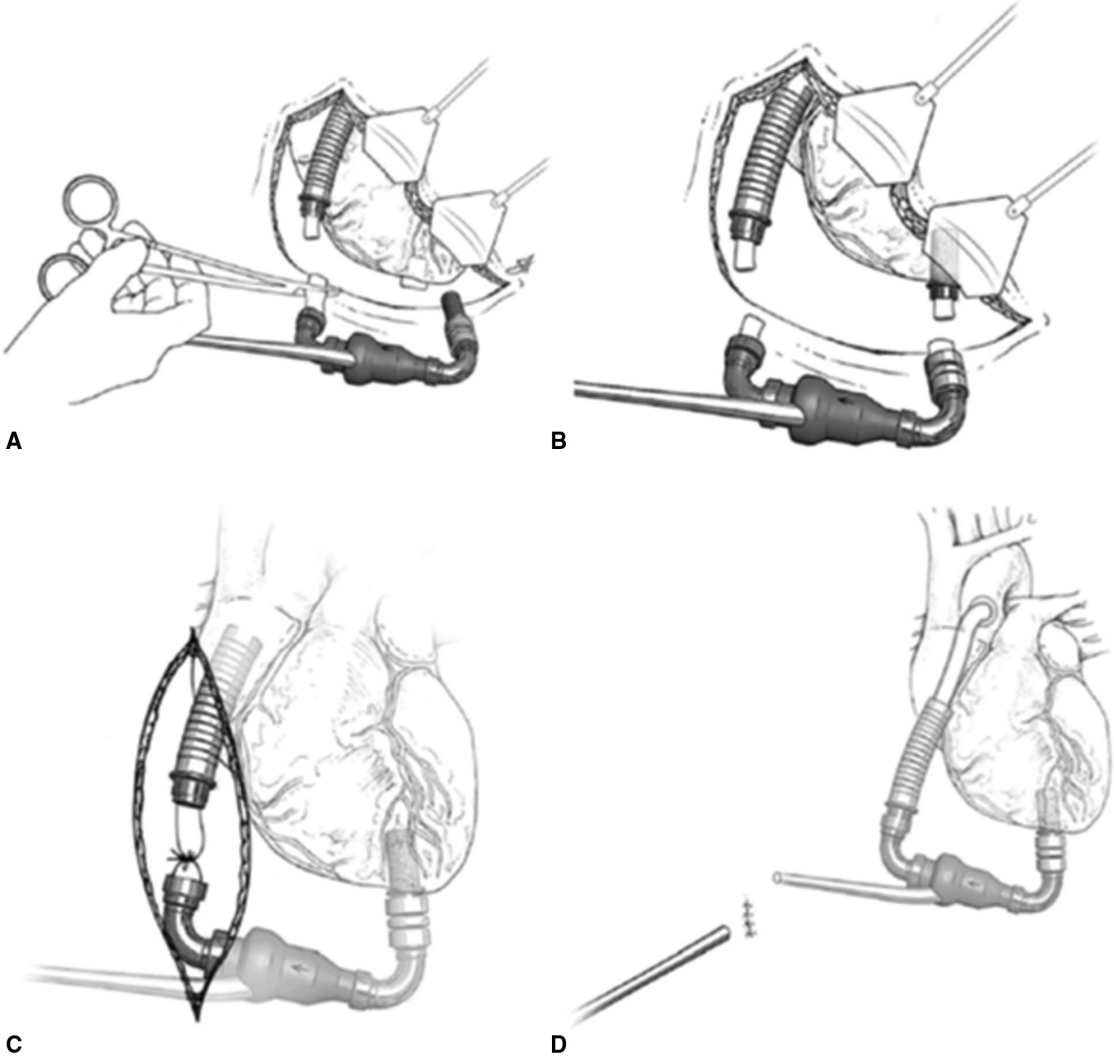
Surgical approaches to HeartMate 2 (Abbott Cardiovascular, Abbott Park, Ill) left ventricular assist device explant. A, Complete pump explant. After removal of the pump housing (including the inlet cannula), the outflow graft is ligated, and the ventricular sewing ring is occluded with a polytetrafluoroethylene plug. B, Inflow and outflow ligation. The inflow bend relief material is excised to allow ligation of the inner graft and retention of the inlet cannula. The pump housing is removed after similar ligation of the outflow graft. C, Outflow graft ligation. The outflow graft is exposed and ligated through a subxiphoid incision. The driveline is then transected at the level of the skin. D, Driveline transection. The pump is left in place, and the percutaneous driveline is cut at the exit site. Reprinted from Baldwin and colleagues.^18^

The HeartMate 2 (Abbott Cardiovascular) device has a unique polyethylene terephthalate inflow graft surrounded by a flexible silicone housing. Incising the housing can expose the graft facilitating ligation. Mobilization of the proximal outflow graft allows for ligation and the pump housing can be removed in its entirety, leaving the apical inflow conduit in situ and the distal outflow graft in the mediastinum. The latter thromboses rapidly and has not been associated with embolic events. We and others have even opted to leave the pump housing intact in the case that the inflow elbow is densely adherent to native tissues. Again, ligation of the inflow graft and outflow graft removes the LVAD from the circulation. In both instances, the driveline is cut as close to the pump housing as possible and pulled through the percutaneous driveline site. In situations where recovery is forced due to pump thrombosis, some authors have simply transected the driveline below the skin and left the entire pump in situ. Figure 2 is from Baldwin and colleagues^18^ and depicts alternative surgical approaches to VAD decommissioning.

Explantation of intrapericardial devices is usually easiest via lateral thoracotomy. The apical cannulation site is directly visualized and the proximal aspect of the outflow graft can be mobilized for ligation. Again, the entire pump housing can be left in situ and the driveline transected at the skin. Authors have also described removing the pump housing and filling the resultant apical defect with custom made plugs. An alternative approach is to perform a right anterior thoracotomy and access the distal end of the outflow graft for ligation. The driveline is transected at the skin and the entire pump housing left in situ without entering the pericardium.

## Conclusions

The ability to enhance myocardial recovery while on mechanical circulatory support remains an elusive goal. Whereas recent evidence from the Remission from Stage D Heart Failure trial does support the concept that targeted pharmacologic therapy can lead to a higher-than-average rate of LVAD explant. Most reports have confirmed that patients with dilated cardiomyopathy can display sustained recovery of myocardial function after LVAD explant.

Surgical techniques to facilitate LVAD explant have evolved to pose little perioperative risk, particularly when a device is not infected. Given these data, it is incumbent upon LVAD caregivers to enhance native myocardial recovery with appropriate medical therapy and to continually assess their supported patients for evidence of recovery.

### Conflict of Interest Statement

Dr Rao is a consultant to Medtronic and Abbott and receives speakers fees from both companies and serves on the North American Surgical Advisory Board to Medtronic. The other author reported no conflicts of interest.

The *Journal* policy requires editors and reviewers to disclose conflicts of interest and to decline handling or reviewing manuscripts for which they may have a conflict of interest. The editors and reviewers of this article have no conflicts of interest.

## References

[1] Birks E.J., Tansley P.D., Hardy J., George R.S., Bowles C.T., Burke M. (2006). Left ventricular assist device and drug therapy for the reversal of heart failure. N Engl J Med.

[2] Molina E.J., Shah P., Kiernan M.S., Cornwell W.K., Copeland H., Takeda K. (2021). The Society of Thoracic Surgeons Intermacs 2020 annual report: the changing landscape of devices and indications. Ann Thorac Surg.

[3] Posada J.G.D., Moayedi Y., Alhussein M., Rodger M., Alvarez J., Wintersperger B.J. (2017). Outflow graft occlusion of the HeartMate 3 left ventricular assist device. Circ Heart Fail.

[4] Maybaum S., Mancini D., Xydas S., Starling R.C., Aaronson K., Pagani F.D. (2007). Cardiac improvement during mechanical circulatory support. A prospective multicenter study of the LVAD Working Group. Circulation.

[5] Yau T.M., Pagani F.D., Mancini D., Chang H.L., Lala A., Woo Y.L. (2019). Intramyocardial injection of mesenchymal precursor cells and successful temporary weaning from left ventricular assist device support in patients with advanced heart failure: a randomized clinical trial. JAMA.

[6] Birks E.J., Drakos S.G., Patel S.R., Lowes B.D., Selzman C.H., Starling R.C. (2020). Prospective multicenter study of myocardial recovery using left ventricular assist devices (RESTAGE-HF [remission from stage D heart failure]): medium-term and primary end point results. Circulation.

[7] Imamura T., Nguyen A., Kim G., Raikhelkar J., Sarswat N., Kalantari S. (2019). Optimal haemodynamics during left ventricular assist device support are associated with reduced haemocompatibility-related adverse events. Eur J Heart Fail.

[8] Delgado D.H., Rao V., Miriuka S.G., Al-Hesayen A., McIver J., Feindel C.M. (2004). Explantation of a mechanical assist device: assessment of myocardial recovery. J Card Surg.

[9] Luo M., Anderson M.E. (2013). Mechanisms of altered Ca^2^+ handling in heart failure. Circ Res.

[10] Flesch M., Margulies K.B., Mochmann H.C., Engel D., Sivasubramanian N., Mann D.L. (2001). Differential regulation of mitogen-activated protein kinases in the failing human heart in response to mechanical unloading. Circulation.

[11] Burkhoff D., Topkara V.K., Sayer G., Uriel N. (2021). Reverse remodeling with left ventricular assist devices. Circ Res.

[12] Zafeiridis A., Jeevanandam V., Houser S.R., Margulies K.B. (1998). Regression of cellular hypertrophy after left ventricular assist device support. Circulation.

[13] Kinoshita M., Takano H., Takaichi S., Taenaka Y., Nakatani T. (1996). Influence of prolonged ventricular assistance on myocardial histopathology in intact heart. Ann Thorac Surg.

[14] Razeghi P., Sharma S., Ying J., Li Y.P., Stepkowski S., Reid M.B. (2003). Atrophic remodeling of the heart in vivo simultaneously activates pathways of protein synthesis and degradation. Circulation.

[15] Dipla K., Mattiello J.A., Jeevanandam V., Houser S.R., Margulies K.B. (1998). Myocyte recovery after mechanical circulatory support in humans with end-stage heart failure. Circulation.

[16] Gupte A.A., Hamilton D.J., Cordero-Reyes A.M., Youker K.A., Yin Z., Estep J.D. (2014). Mechanical unloading promotes myocardial energy recovery in human heart failure. Circ Cardiovasc Genet.

[17] Margulies K.B., Matiwala S., Cornejo C., Olsen H., Craven W.A., Bednarik D. (2005). Mixed messages: transcription patterns in failing and recovering human myocardium. Circ Res.

[18] Baldwin A.C.W., Sandoval E., Letsou G.V., Mallidi H.R., Cohn W., Frazier O.H. (2016). Surgical approach to continuous-flow left ventricular assist device explantation: a comparison of outcomes. J Thorac Cardiovasc Surg.

